# Differentially Expressed Salivary Proteins in Dental Caries Patients

**DOI:** 10.1155/2021/5517521

**Published:** 2021-10-14

**Authors:** Zaid Majeed Khan, Humera Waheed, Zohaib Khurshid, Muhammad Sohail Zafar, Syed Faraz Moin, Mohammad Khursheed Alam

**Affiliations:** ^1^National Center for Proteomics, University of Karachi, Karachi 75270, Pakistan; ^2^Dow College of Biotechnology, Dow University of Health Sciences, Karachi 75270, Pakistan; ^3^Department of Prosthodontics and Dental Implantology, College of Dentistry, King Faisal University, Al Ahsa 31982, Saudi Arabia; ^4^Department of Restorative Dentistry, College of Dentistry, Taibah University, Al Madinah, Al Munawwarah 41311, Saudi Arabia; ^5^Department of Dental Materials, Islamic International Dental College, Riphah International University, Islamabad, Pakistan; ^6^Preventive Dentistry Department, College of Dentistry, Jouf University, Saudi Arabia

## Abstract

Dental caries is a multifactorial disease mainly caused by cariogenic bacteria commonly found in the oral cavity. Dental caries may cause demineralization of the tooth, cavitation, hypersensitivity, pulp inflammation, and even tooth loss if left untreated. Saliva secreted in the oral cavity can serve as a tool for identification of biomarkers for early detection of diseases. In the present study, differential expression of salivary proteins from 33 dental caries patients was compared with 10 control subjects. The unstimulated saliva was analyzed by 12% SDS-PAGE and two-dimensional gel electrophoresis. Gelatin and casein zymography was performed to check for protease activity. Also, salivary IgAs from both groups were compared by sandwich ELISA technique. Dental caries patient's saliva showed decreased caseinolytic and increased gelatinolytic activity probably due to metalloproteases and cathepsins. Mean salivary levels of sIgA were also significantly higher (*p* < 0.018) in dental caries saliva samples. The 2D electrophoresis profile of both the groups showed regions on gel with visually detectable alterations in protein expression. The present study is among the few initial studies in the locality for identification of protein differences in saliva from dental caries patients and has demonstrated a good potential to identify alterations. However, a large population-based analysis is required to validate these findings to be translated as a tool for indicative applications.

## 1. Introduction

Dental caries (tooth decay) is a common oral condition cause by acids produced by bacteria resulting in dissolution of tooth surface. It is a multifactorial and highly prevalent disease that is related to unhealthy lifestyle of a person. There are about 3.5 billion cases related to oral conditions of which about 2.3 billion are those related to permanent teeth while 532 million cases of dental caries related to primary teeth [1, 2]. Tooth decay is caused by a complex interaction of cariogenic bacteria residing in dental biofilm (plaque) that ferment dietary carbohydrates, produce an acidic pH, and result in demineralization and cavitation. If the condition remains untreated, then it spreads to pulp causing pain and finally leads to tooth loss [3, 4]. The key etiological factors include high consumption of sugary food and beverages, poor hygiene, low salivary function, and fluoride deficiency. Factors like social class, geographical location, race, age, and sex are also influential in developing caries [5, 6].

Saliva is secreted mainly from three major and several minor salivary glands. Whole mouth saliva (WMS) consists of water, proteins, peptides, electrolytes, minerals, and microorganisms which play an important role in saliva function and maintaining oral homeostasis [7, 8]. In addition, saliva aids in lubrication, speech, mastication, digestion of food, taste sensing, wound healing, and overall protection of teeth and the oral cavity [9]. The protein concentration of saliva is around 2.0 mg/mL [10]. However, under various systemic and pathological conditions, the concentration of salivary protein expression may be altered. Despite small proportion, salivary proteins may play a protective or an unprotective role in the oral cavity. For example, lactoferrin, peroxidases, and lysozyme act as cariogenic bacterial inhibitor and modulators of the mineralization and demineralization process [11]. Other salivary peptides such as histatins, defensins, statherin, and cathelicidins control oral flora and thus serve a protective role [12, 13]. On the other hand, certain proteins have shown to have cariogenic roles by promoting colonization and proliferation of oral microbes. For instance, common salivary protein-1 can bind to Streptococcus mutans and enhances its adherence to salivary pellicle formed on hydroxyapatite surfaces suggesting its cariogenic role by promoting bacterial colonization on the surface of the tooth [14]. Besides, the role of many salivary proteins particularly in disease pathogenesis is still obscure. The main reason is that most of the functional elucidation of salivary proteins is obtained through classical proteomics and biochemical analysis. High-throughput proteomics studies and tools may comprehensively help in the characterization and functional translation of all salivary proteins [15].

It is evident that WMS reflects the physiological status of the oral cavity and the whole body. A study of drug monitoring following the model of transmembrane transport had shown that many of the saliva constituents are released by active transport, diffusion, and extracellular ultrafiltration from glands, blood, serum, and tissues of the oral cavity [16]. The significance of saliva as a diagnostic tool comes from the fact that it involves a safer, noninvasive, inexpensive collection process that requires minimal processing and trained workers. Also, multiple samples can be collected easily with minimal infection risk and cold storage conditions [17]. Most of the oral pathological conditions can be prevented or have decreased severity and increased therapy success if detected at an early stage. There is abundance of components in saliva that could be used as biomarkers for diagnosis, prognostication, treatment planning, and posttherapy monitoring for both local and systemic diseases [18]. For example, various factors such as salivary flow rate, its buffering capacity, and other constituents are associated with caries risk [19]. The main antibody in saliva is secretory immunoglobulin A (sIgA) with small fractions of other antibodies. The concentrations of these antibodies keep changing with age and are independent on gender [20]. The sIgA provides immunity by inhibition of microbial adhesion, toxins, and enzyme neutralization and by reducing hydrophobicity in synergism with lactoferrin and lysozymes [21]. Studies conducted on children suggest increased susceptibility to dental caries due to deficiency of sIgA and increased expression during caries suggesting a protective role; however, there is contradiction on correlation between dental caries and sIgAs [22–24]. In addition, matrix metalloproteinases (MMPs) and cathepsins are associated with dental caries progression and pathogenesis. MMPs are produced by odontoblasts that promote dentin formation and become entrapped in calcified matrix during collagen matrix mineralization process. Under acidic environment, the entrapped MMPs become activated and destroy matrix components [25, 26]. Among the members of MMP family, MMP-8 and MMP-9 in dentin and saliva are mainly associated with proteolytic activity and thus promote dentin degradation [27].

Therefore, the current study was conducted to compare the differential expression of proteins in saliva of normal and dental caries patients. According to the best of authors' knowledge, the present study is the first one reporting the use of casein zymography for salivary protease analyses and also the first report of saliva proteins in caries patients of local population.

## 2. Material and Method

### 2.1. Patient Recruitment and Saliva Processing

The present study recruited a total of 33 dental caries and 10 control patients (caries-free at the time of sample collection, no lesions) according to the convenience (nonprobability) sampling method. The sample size was kept according to suitability to easily collect the data. The study participants (age: 20-50 years of either sex) were in good overall general health with no history of smoking or pan/gutka chewing, diabetes, or any other oral/systemic disease for at least last 3 months. The dental caries status of all the participants was analyzed according to the International Caries Detection and Assessment System (ICDAS) during the clinical examination between 9 AM and 11 AM by a dentist in clinical practice in the city of Karachi, Pakistan. After the clinical examination, patients were asked for a gentle mouth rinsing with water, and unstimulated saliva (a minimum of 2 mL) was collected by a passive drooling method in sterile polypropylene tubes on ice. Participants abstained from eating or taking medication at least 2 hours prior to sample collection. Samples were cold centrifuged for 15 min at high speed (7000 rpm) to remove any solid particles from the oral cavity. The supernatant was separated and stored at low temperature (-20°C) until further analysis. This study was approved by the Institutional Ethical Committee, University of Karachi (IBC KU-94/2020), and an informed consent was obtained from each participant.

### 2.2. Protein Estimation

Total salivary protein concentration was estimated through the bicinchoninic acid (BCA) protein estimation kit (Thermo Scientific™ Pierce™) as instructed by the manufacturer [28]. Bovine serum albumin (BSA) protein was used to develop the standard curve, and the absorbance was recorded at 562 nm [29].

### 2.3. Protein Analysis by Sodium Dodecyl Sulfate-Polyacrylamide Gel Electrophoresis (SDS-PAGE)

The proteins in individual and pooled saliva samples were analyzed in reducing and nonreducing conditions on 12% SDS-PAGE according to the procedure described by Laemmli [30]. Electrophoresis was performed for 2 hours at constant 70 V. After completion, gels were fixed overnight in gel fixing solution (50% methanol, 6% acetic acid, and 0.025% formaldehyde). Gels were then washed 3x using the deionized distilled water. Protein bands were visualized by staining with Coomassie Brilliant Blue R-250 (0.02% *w*/*v*).

### 2.4. Zymographic Analysis

The protease activity in dental caries patients and controls (individual and pooled saliva samples for increased resolution and sensitivity) was analyzed by casein and gelatin zymography. Saliva samples were run on to 12% polyacrylamide gels copolymerized with either 0.3% gelatin or casein as a protease substrate. Previously, casein was solubilized in 10 mM Tris-HCl (pH 8.8) while gelatin was simply solubilized in deionized water at 37°C. After electrophoresis, gels were washed with wash buffer (Tris-HCl, 2.5% Triton X-100) twice with constant shaking for 30 min to remove SDS. Gels were placed in an activation buffer (Tris-HCl, calcium chloride, and Triton X-100) overnight at 37°C. The next day, gels were visualized after staining with Coomassie Blue and destaining with deionized water.

### 2.5. 2-Dimesional Electrophoretic Analysis

The differential expression of proteins in both the groups was mapped by 2D gel electrophoresis (PROTEAN IEF system, Bio-Rad Laboratories, USA) as described previously [31]. Briefly, the pooled saliva (80 *μ*g) from dental caries and control groups was mixed in rehydration buffer and loaded on to immobilized pH-gradient (7 cm, pH 3-10; Bio-Rad) gel strips. Strips were layered with mineral oil and rehydrated for 14-16 hours at room temperature. The next day, isoelectric focusing (IEF) was performed as follows: 200 V (1 min), 3000 V (1.5 hrs), and 3500 V (3 hrs) to reach 12000 V/hr. The IPG strips were reduced in equilibration buffer (50 mM Tris-HCl-pH 8.8, 6 M urea, 2% SDS, 30% glycerol, and 10 mg/mL DTT) for 30 minutes followed by alkylation (0.5 M Tris-HCl pH 6.8, 12 M Urea, 10% SDS, 60% glycerol, and 25 mg/mL iodoacetamide) for 30 minutes. The proteins were then separated based on their molecular weight in second dimension on 12% SDS-PAGE gels and visualized by Coomassie Brilliant Blue R-250.

### 2.6. Gel Analysis

Differential expression patterns of proteins in SDS-PAGE, zymogram, and 2D gels from diseased (caries) and control gels were analyzed via gel analysis software (PD Quest, Version 8.0, Bio-Rad). Also, in zymography, gel band intensities in both groups were measured with Bio-6000 gel scanner (from Bioimager, Canada) and were compared.

### 2.7. Secretary IgA (sIgA) Analysis by ELISA

Salivary secretory IgA levels of both the groups were analyzed by sandwich ELISA by using ELISA Kit (Catalog No. E-EL-H1275 96T). Experiment was performed according to the manufacturer's instructions. Each sample was analyzed in duplicate, and the absorbance was recorded at a wavelength of 450 nm. Secretory IgA concentration was determined from a calibration curve established from a standard antibody provided with the kit. The sIgA concentration between control and diseased (caries) groups was compared statistically by using Student's *t*-test (unpaired). The *p* value of <0.05 was considered significant.

## 3. Results

### 3.1. SDS-PAGE Reducing and Nonreducing Condition

**Figure 1:**
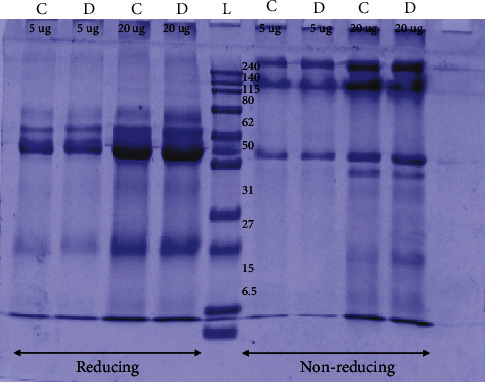
SDS-PAGE analysis of saliva: pooled saliva samples of control (C) and diseased (caries) (D) patients were run using 12% SDS-PAGE gels. The concentration of saliva loaded in each well starting from left to right is 5 *μ*g lanes 1 and 2, C and D, respectively, and 20 *μ*g lanes 3 and 4, C and D, respectively, under reducing conditions. Lane 5 is molecular weight marker. Lanes 6 and 7, 5 *μ*g C and D, respectively, and lanes 8 and 9, 20 *μ*g C and D, respectively, under nonreducing conditions.

The salivary analysis of pooled control and dental caries groups on SDS-PAGE showed similar results under reducing and nonreducing conditions. The clear protein bands ranging from >240 kDa to <6.5 kDa were observed (Figure 1). However, the individual samples from both groups showed variation in low molecular weight bands that are easily observed and more in number in diseased (caries) group as compared to the control group (data not shown). It has been reported earlier that the apparent molecular weight, number, and intensity of the bands vary from individual to individual due to genetic phenotypic polymorphism [32]. Mainly, mucins and proline-rich protein (PRP) vary due to certain factors like age, oral hygiene, and oral conditions such as caries. Mucins are 200-100 kDa molecular weight proteins while PRPs such as PRP1 present in saliva is a 30 kDa protein [33, 34]. Our results of pooled samples also showed the same pattern of bands in control and diseased (caries) groups irrespective of the concentrations loaded on to the gel. The results reported by Schwartz et al. of resting pooled saliva where the individual saliva samples were also analyzed present the same observations on SDS-PAGE as were obtained during this study [35].

### 3.2. Zymography of Saliva Samples

**Figure 2:**
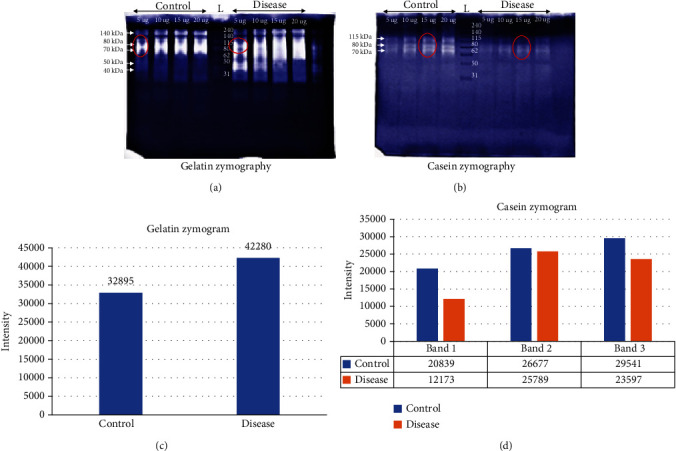
Zymographic analysis of saliva: (a) gelatin zymogram and (b) casein zymogram of pooled saliva sample of controls and dental caries patients. Lanes left to right showed increasing concentration of controls and diseased (caries) samples. On the left side of each gels, apparent molecular weights of proteases were mentioned. (c) Bar graph for protease activity area comparison on the basis of band intensity of gelatin zymogram (80 kDa band) and (d) casein zymogram (115, 80, 70 kDa, respectively). Red circles represent activity regions used for intensity. (L) Molecular weight marker.

The proteolytic activity of two groups was compared by gelatin (Figure 2(a)) and casein (Figure 2(b)) zymography performed on individual and pooled saliva samples. The individual samples showed wide diffused bands of activity throughout the respective lanes in gel even with low concentrations (data not shown). However, the pooled samples showed increased band resolution on both zymographic gels. Visual analysis showed an increase in the proteolytic activity by increasing the sample concentration. In dental caries patients, increased gelatinolytic activity and decreased caseinolytic activity were observed in comparison to controls (Figure 2). This proteolytic activity may be associated with increased proteases such as cathepsins and metalloproteases. The apparent molecular weight of different proteases was ~140, 80, 70, 50, and 40 kDa in the gelatin gels (Figure 2(a)) and ~115, 80, and 70 kDa (Figure 2(b)) in casein gels. In gelatin zymogram, activity bands at a molecular weight of ~40 and 50 kDa were not observed in control saliva samples. Also, diffused bands were observed in gelatin zymogram probably due to increase protease activity and close proximity of molecular weight of the enzymes.

The band intensities of selected bands (Figures 2(a) and 2(b)) on gelatin and casein of controls and diseased were analyzed by using MAX 10000 imager software. The area of intensity was analyzed (Figures 2(c) and 2(d)). The data also supports increase in protease activity in the dental caries group compared to the controls in gelatin zymographs. In casein zymography, band at 115 and 70 kDa is more intense in controls (band 1 and 3) as compared to dental caries group. However, band at 80 kDa (band 2) showed slight difference in intensity (Figure 2(d)).

### 3.3. 2D Gel Electrophoresis

**Figure 3:**
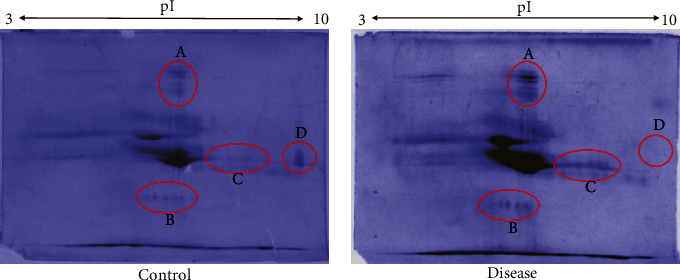
2D gel electrophoretic profile of saliva: differential protein expression of pooled saliva from control and dental caries patients. Red circles labelled A, B, C, and D show proteins differentially expressed in both groups.

The differential proteome map of pooled saliva samples of both the groups was obtained by 2D gel electrophoresis (Figure 3). For isoelectric focusing, strips of pH gradient (3-10) were used followed by 12% SDS-PAGE. Gels were visualized, and images were taken by using PD Quest software. Most of the protein's spots were not resolved due to spot clustering caused by higher concentration of amylase and immunoglobulins in saliva (dark patches). However, a few proteins showed differential expression among both the groups. Proteins spots in circles A, B, and C showed higher expression in the diseased (caries) group while circle D showed proteins that were only expressed in controls.

### 3.4. Detection of IgAs by ELISA

**Figure 4:**
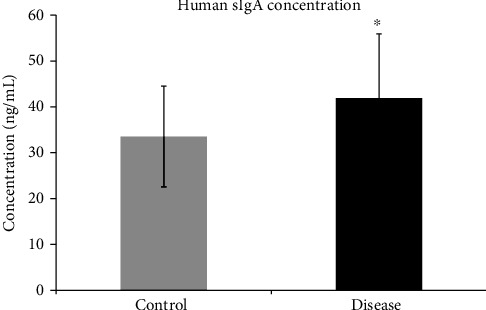
sIgA detection by ELISA: concentration of human sIgA is specified as mean ± SD. Analysis of the data was performed by Student's *t*-test (unpaired). A result with *p* values < 0.05 was considered statistically significant. Asterisk (∗) indicates statistically significant difference.

Sandwich ELISA was used to compare salivary levels of sIgAs among both the groups in nanograms per milliliter. The mean salivary IgA levels were found to be high in caries patient while low levels were detected in the control group (Figure 4). Student's *t*-test (unpaired) showed the significant differences with a *p* value equal to 0.018 which is less than 0.05 (standard cut-off value to determine significant differences among groups). The mean ± SD sIgA levels were found to be 41.89 in the dental caries and 33.53 ng/mL in the control group.  Table 1 also represents the lowest and highest values of sIgA obtained in both group samples.

## 4. Discussion

Saliva is an emerging source for disease biomarker identification and analysis due to its stress-free, simple, noninvasive collection methods [31, 36]. Any pathological or abnormal condition results in alteration of salivary composition. This makes saliva as one of the most important sources where biomarkers for the existence and advancement of dental problems may be explored. Bacteria are the main causative agents associated with caries formation and therefore activate the host immune system to release antimicrobial agents in saliva including immunoglobulins like IgA and IgG, lactoferrin, lysozyme, hypothiocyanite, peroxidase/myeloperoxidase, and agglutinins [37].

The present study analyzed the differential expression of salivary proteins from dental caries patients and healthy individuals. For this purpose, the saliva samples of participants were analyzed using various analytical techniques including SDS-PAGE, zymography, and sandwich ELISA. The present study revealed decreased caseinolytic and increased gelatinolytic activity in the diseased (caries) group likely due to metalloproteases and cathepsin proteins. Bacterial and host proteases (from dentin and saliva) are altogether responsible for dentin extracellular matrix degradation in carious decay process. Saliva contains gelatinases (MMP-2, MMP-9), collagenases (MMP-1, MMP-8, and MMP-13), enamelysin (MMP-20), and cysteine cathepsins of various types [27, 38, 39]. Bacterial acids promote demineralization, degrades SIBLINGs (Small Integrin-binding Ligand N-linked Glycoproteins), and activates host pro-MMPs to digest dentin matrix. MMP-2, MMP-9, and MMP-8 have been detected from carious lesion by gelatin zymography and Western blot analysis [40]. In addition, increased expression of MMP-13 has been observed in caries dentin by immunohistochemical analysis [41]. On the other hand, cathepsins (B and L) under mild acidic conditions initiate matrix degradation, activate pro-MMPs, and degrade native type I collagen [42]. In our study, increase in protease activity was observed in dental caries patients which may be associated with increased expression of MMPs and cathepsins. Contrary to gelatin zymography, an increased caseinolytic activity in the control group was observed compared to the diseased (caries) group while overall protease activity is less as observed on gelatin gels. This may be attributed to the difference in source of two substrates, i.e., gelatin (derived from collagen) and casein (obtained from milk). Casein is a nonspecific substrate and is less sensitive for proteases particularly collagenases [43, 44]. The use of casein zymography for salivary protease analyses is so far the first report as no other study has been found utilizing casein zymography for saliva protease analysis.

Secretory IgAs are predominant immunoglobulin found in saliva and act as the first line of defense against infectious nature of cariogenic microorganisms. sIgAs inhibit not only microbial attachment but also their toxins by immune exclusion mechanisms. It also protects the epithelial surfaces by controlling symbiotic association between host and commensals [45]. Secretary IgAs are also found to interrupt certain virulence factors and cause decrease in inflammation by controlling cytokine and interleukin response [46]. Analysis of dental caries and control saliva samples for sIgA detection by ELISA showed significant increased expression in the caries group (*p* value < 0.018). On the basis of molecular mass, increased expression of immunoglobulins was observed in the two-dimensional (2D) gels (Figure 3, circle A) as more intense spots were noted at the resolution point of sIgAs according to Ghafouri et al. [47]. Similar to our findings, de Farias and Bezerra and Thaweboon *et al.* also stated that dental caries is associated with increased levels of salivary sIgA [48, 49]. Contrary to our study, others observed an inverse relationship between salivary sIgAs and dental caries [50]. At the same time, a few studies did not find any association between the two [51, 52]. These contradictory observations may be attributed to different saliva sampling methods, exposure to different environmental factors, and inclusion criteria used for subject selection. Also, salivary IgA levels are affected by age, pregnancy, presence of any other systemic disease, salivary flow rate, use of medications, hormonal changes, and physical activity. Therefore, studies using standardized sample collection and large sample size must be carried out to clearly establish sIgA role in dental caries [53].

The comparison of 2D gels of both groups showed four regions (encircled in red) in Figure 3 where differential protein expression in caries was observed. Among those, circles A, B, and C indicate the increased expression in disease condition (Figure 3, circles A, B, and C) whereas circle D showed downregulation (Figure 3, circle D). These highly expressed protein in the caries group could be attributed to many of the immune factors and other protein constituents of saliva. These include salivary IgAs (expected Figure 3, circle A), cystatins (expected Figure 3, circle B), and proline-rich proteins (expected Figure 3, circle C) as reported in salivary proteome analysis on 2D gels [47, 54, 55]. A dense band observed at the center for gel is expected to be salivary albumin, amylases, and immunoglobulins that interact with each other and other proteins to form an interactome. The other factor for such an observation could be the pooling of samples that may have enhanced the interactome effect of the sample. Moreover, it has been reported that almost 66 different proteins were found to interact with amylases only. This suggests its additional role in transport and protection of its partner proteins [56]. Cystatins and proline-rich proteins along with statherin inhibit bacterial and viral pathogens to maintain salivary calcium levels, enhance remineralization, and thereby serve a protective role in maintaining teeth integrity [57]. This could also be an element to increased expression of protein spots observed in dental caries patients indicating host immune response to combat the disorder. However, mass spectrometric analysis of salivary 2D gels is further required to accurately identify the presence and upregulation of these proteins. The need to look into the posttranslational modifications such as the phosphoproteome and glycoproteome of salivary proteins would be of great interest and a side that is lacking. Specifically, the glycoproteome studies would provide excessive support in this regard in salivary diagnostics [58].

## 5. Conclusions

Dental caries is the most prevalent oral disease that involves demineralization and breakdown of dental hard tissue in the presence of acidic environment produced by oral microbes. The protein profiling of control and diseased saliva samples on SDS-PAGE and 2D-electrophoresis showed certain expressional differences. However, proteolytic activity and mean sIgA levels were significantly higher in caries active patients. The present study is among the initial studies for identification of salivary proteins in dental caries patients in the local community. Our analysis has demonstrated a good potential to identify protein differences due to dental caries.

## Figures and Tables

**Table 1:** Comparison of salivary secretory immunoglobulin A (sIgA) in control and dental caries patients in nanograms per milliliter.

Groups	Lowest value	Highest value	(mean ± SD)
Control	13.8	49.0	33.53 ± 11.59
Disease	7.47	67.7	41.89 ± 14.82

## Data Availability

The data used to support the findings of this study are included within the article.

## Abbreviations

ELISA: Enzyme-linked immunosorbent assay
2D-electrophoresis: Two-dimensional electrophoresis
SDS-PAGE: Sodium dodecyl sulfate-polyacrylamide gel electrophoresis
PRP: Proline-rich proteins
sIgAL: Secretory immunoglobulin A
MMP: Matrix metalloproteinase
BSA: Bovine serum albumin
BCA: Bicinchoninic acid
IPG Strips: Immobilized pH gradient strips
IEF: Isoelectric focusing.
